# Expanding the landscape of the unfolded protein response: The roles of secondary transcription factors in development and disease

**DOI:** 10.1016/j.cstres.2025.100141

**Published:** 2025-12-18

**Authors:** Miguel Angel Jiménez-Beltrán, Rocío Valle-Bautista, Edgar Ricardo Vázquez-Martínez

**Affiliations:** Unidad de Investigación en Reproducción Humana, Instituto Nacional de Perinatología (INPer) - Facultad de Química, Universidad Nacional Autónoma de México (UNAM), Mexico City 11000, Mexico

**Keywords:** UPR, Transcription factor, XBP1, ATF4, ATF6

## Abstract

The unfolded protein response (UPR) of the endoplasmic reticulum (ER) is a classic cellular reaction to stress that helps restore ER homeostasis. However, growing evidence demonstrates that the main UPR effectors (Activating Transcription Factor 6 (ATF6), X-box Binding Protein 1 (XBP1s), and Activating Transcription Factor 4 (ATF4)) not only regulate canonical UPR target genes but also promote the transcription of genes encoding secondary transcription factors (TFs). These secondary TFs contribute to ER homeostasis maintenance and are involved in various physiological processes that extend beyond the traditional UPR. In this review, we examine the secondary TFs activated by UPR master regulators (UPR-TFs) and discuss their functional roles in different tissues and organs. We emphasize how these secondary TFs, controlled by their respective UPR-TFs, participate in stress responses, cell differentiation, embryonic development, circadian rhythms, metabolism, and other physiological processes. Furthermore, we explore common signaling pathways and tissue- and cell-specific regulatory mechanisms, highlighting convergence points where secondary TFs from different UPR branches intersect, indicating a more complex regulatory network. We also discuss the functions of these secondary TFs in the lungs, placenta, testis, uterus, pancreas, and liver, as well as during embryonic development and in pathological conditions. This study reveals biological activities that extend beyond the traditional roles of the UPR, providing a broader view of this signaling pathway and opening new avenues for future research.

## Introduction

When exposed to stress, cells can trigger temporary adaptive responses aimed at repairing damage and maintaining cellular function, thereby promoting cell survival. However, if the stress persists, it may cause irreversible cellular damage, leading to senescence or programmed cell death, which preserves tissue homeostasis and organismal survival.^1^ One of the most extensively studied organelles involved in maintaining cellular homeostasis is the endoplasmic reticulum (ER).^2^ Its roles within the cell have been widely investigated, particularly in lipid and steroid metabolism, gluconeogenesis, proteostasis, and calcium storage.^3^, ^4^

The ER participates in a wide range of cellular processes, establishing it as a highly dynamic organelle that is sensitive to both intra- and extra-cellular fluctuations. Under physiological conditions, the ER maintains protein homeostasis, which is essential for cell survival and proper function. This is because the ER lumen contains chaperones responsible for the correct folding of newly synthesized proteins, such as calnexin and calreticulin.^5^ Under stress conditions such as oxidative stress, starvation, and hypoxia^6^ chaperones may become dysfunctional or overwhelmed, resulting in the accumulation of misfolded proteins within the ER lumen (a condition termed ER stress), which subsequently triggers the unfolded protein response (UPR).^7^, ^8^

UPR induction requires the participation of three main effectors that are activated upon dissociation of the molecular chaperone glucose-regulated protein 78 (GRP78) in response to accumulating unfolded proteins: protein kinase R-like endoplasmic reticulum kinase (PERK), inositol-requiring enzyme 1 alpha (IRE1α), and activating transcription factor 6 (ATF6). These proteins coordinate transcriptional and translational programs aimed at restoring proteostasis through enhanced protein folding capacity via ER-associated degradation (ERAD), lysosomal degradation, and autophagy. If homeostasis cannot be reestablished, programmed cell death is activated.^9^

Dysregulation of the UPR has been implicated in various pathological conditions, including cancer, where disruption of this homeostatic mechanism modulates critical processes such as inflammation, angiogenesis, and immune evasion.^10^, ^11^, ^12^ Consequently, aberrant activation or deactivation of the UPR can promote the survival of malignant cells.^13^ A particularly significant finding is the consistent upregulation of the master initiator of the UPR signaling, GRP78, across diverse cancer types.^14^ This observation has prompted extensive research into the role of UPR components in tumor development, particularly in colorectal carcinoma,^15^ glioblastoma^16^ and pancreatic cancer.^17^ Although this is a highly significant area of biomedical research, it lies beyond the scope of the present review. We refer readers to excellent recent reviews that address this important topic in detail.^11^, ^12^

Regardless of the experimental model or physiological condition, UPR signaling pathway activation requires well-coordinated communication between the ER and the nucleus. This intimate crosstalk underscores the pivotal role of the ER in regulating gene expression programs, particularly through the regulation of transcript synthesis and localization during ER stress.^18^

Recent years have witnessed a substantial increase in studies investigating transcriptional programs that restore proteostasis in response to ER stress induction and UPR activation. However, information regarding the cooperative interactions between the primary transcription factors (TFs) involved in the UPR (ATF6, activating transcription factor 4 (ATF4), and X-box binding protein 1 (XBP1s)), here collectively designated as UPR-TFs, and the transcriptional regulation of secondary TFs synthesized downstream (here referred to as secondary TFs), remains poorly explored. Moreover, these secondary TFs may play broader roles beyond canonical ER stress responses. This review examines the regulatory mechanisms and potential cooperative functions of these secondary TFs in coordination with UPR-TFs, emphasizing their functional significance in physiological and pathological conditions.

## UPR activation and its transcriptional axes

UPR activation is typically triggered by the accumulation of unfolded and misfolded proteins within the ER lumen. Under basal conditions, the molecular chaperone GRP78 binds to the luminal domains of the transmembrane sensors PERK, IRE1α, and ATF6, maintaining them in an inactive state. However, when unfolded and misfolded protein levels increase, GRP78 dissociates from these sensors to preferentially bind the exposed hydrophobic regions of misfolded proteins.^19^, ^20^, ^21^

Upon GRP78 dissociation, PERK and IRE1α undergo dimerization and autophosphorylation, leading to their activation.^22^, ^23^ In contrast, ATF6 dissociation enables its packaging into coat complex protein II (COPII)coated vesicles for transport to the Golgi apparatus, where two sequential proteolytic cleavages release the active cytosolic domain that functions as a TF.^24^, ^25^

Once activated, PERK promotes the selective translation of ATF4, which possesses TF activity. Simultaneously, IRE1α exhibits endonuclease activity that splices XBP1 mRNA, generating the spliced isoform XBP1s, which is subsequently translated into a functional TF.^26^

### UPR and its transcriptional extensions: From master regulators to secondary TFs

**Fig. 1:**
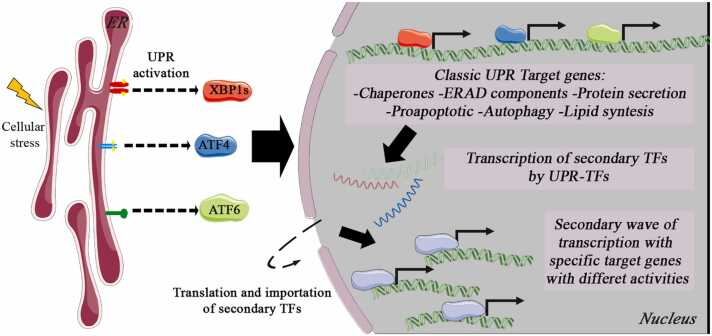
*The unfolded protein response (UPR) and its downstream transcriptional cascade.* Following ER stress, the three main UPR branches activate their effector transcription factors (XBP1s, ATF4, and ATF6, collectively referred to as UPR-TFs), which regulate canonical target genes that restore homeostasis, including chaperones, ERAD components, autophagy-related proteins, and lipid synthesis regulators. Concurrently, these UPR-TFs also promote the transcription of genes encoding secondary transcription factors (TFs). Once translated and translocated to the nucleus, these secondary TFs trigger a second wave of transcriptional programs, controlling distinct gene sets involved not only in ER stress adaptation but also in broader physiological pathways and tissue-specific processes.

The activation of the UPR triggers a tightly regulated transcriptional program orchestrated by the three primary TFs (ATF4, ATF6, and XBP1s). While these master regulators have been extensively studied for their canonical roles in restoring ER homeostasis, recent evidence reveals that their transcriptional influence extends far beyond the classical stress response. This section explores the individual pathways of each UPR-TFs, the regulation of downstream secondary TFs, and the broader cellular processes influenced by these extended networks (Figure 1). To identify the secondary TFs listed in Table 1, Table 2, Table 3, we conducted a comprehensive literature search in PubMed. We included only experimental studies employing high-throughput transcriptomic and chromatin profiling methods, including RNA sequencing (RNA-seq) and chromatin immunoprecipitation sequencing (ChIP-seq). Studies were excluded if they did not provide direct experimental evidence of transcriptional regulation. For each identified TF, we performed a comprehensive literature review to summarize their main biological functions across diverse physiological or pathological contexts, independent of their direct association with UPR signaling. It is important to note that while we report diverse functions for each secondary TF, these activities do not necessarily reflect direct UPR-TF-mediated regulation or occur exclusively during ER stress. Rather, these findings represent the broader functional repertoire of these TFs across multiple biological contexts. Future mechanistic studies will be essential to determine which of these functions are directly controlled by UPR signaling and to define the specific conditions under which such regulation occurs.

**Table 1:** ATF4-regulated secondary TFs and their cellular functions.

Second-ary TFs	Function	Method (used to identify secondary TFs)	Model	Method (used to demonstrate functional role of secondary TFs)	References
DPF2	Regulates mesoderm/endoderm differentiation during embryogenesis, myeloid lineage commitment, and apoptosis. It is part of the chromatin remodeling BRG1/BRM-associated factor (BAF) complex and induces NRF2-dependent anti-inflammatory gene expression via histone mark recognition	Structural/biochemical binding assays, targeted mutagenesis, chromatin recruitment assays, in vivo differentiation assays^27^ Genetic deletion (Dpf2 KO), ChIP/co-occupancy analyses, transcriptomics ^28^ Genetic mouse models Dpf2 KO, transcriptomics, mechanistic/biochemical assays, pharmacological NRF2 reactivation^29^	Myeloid cells/in vivo myeloid differentiation models (mouse)^27^ Mouse embryonic stem cells (ESCs)^28^ Hematopoietic stem cells (HSCs) and immune effector cells; Dpf2 KO mice^29^	Targeted mutagenesis of DPF2 histone-binding pockets, loss of chromatin recruitment and loss of ability to prevent myeloid differentiation in vivo (functional test linking DPF2 chromatin binding to biological effect^27^ Dpf2 deletion (loss-of-function) to test consequence on ESC self-renewal and expression of Tbx3; ChIP/co-occupancy with pluripotency TFs to show regulatory interaction (functional dependence shown by KO; expression/phenotype change)^28^ Genetic deletion of Dpf2 KO and rescue/pharmacological activation of NRF2 to test the DPF2-NRF2 functional axis; phenotypic rescue with NRF2 reactivation demonstrates functional dependence of NRF2-dependent gene expression on DPF2^29^	27, 28, 29
BRF2	Involved in RNA Polymerase III recruitment to type III gene-external promoters; also regulates promoter activity and induces GRP78 expression in acquired middle ear cholesteatoma; module oxidative stress response.	Review: literature synthesis, analysis of expression datasets (Oncomine) linking BRF2 with cancer^30^ Gene expression analyses, IHC in tissue, cell culture (HaCaT) overexpression/KD^31^ Cellular functional assays, mitochondrial homeostasis measurements, apoptosis assays^32^	Multiple cancer cell/tissue datasets (review/data mining)^30^ Human middle ear cholesteatoma tissues; HaCaT keratinocyte cell line^31^ Lung squamous carcinoma cell lines^32^	N/A (review)^30^ Modulation of BRF2 expression in HaCaT cells and measurement of GRP78 expression (overexpression/KD). Gain/loss-of-function in cultured cells to test effect on primary target gene (GRP78)^31^ Perturbation of BRF2 (KD/overexpression) and measurement of apoptosis and mitochondrial homeostasis (SLC8A3-mediated)^32^	30, 31, 32
TAF15	Component of the RNA Polymerase II pre-initiation complex; regulates initiation and elongation splicing factors expression, cell cycle progression, and RNA processing.	Review/synthesis of stress granule biology, LLPS mechanisms; literature compilation^33^ KD (siRNA)/functional proliferation assays, miRNA analyses, gene expression profiling^34^ Immunoprecipitation, in vivo UV cross-linking, pull-down assays, biochemical interaction assays^35^	Multiple neuronal models and cell types reviewed^33^ Cancer/proliferating cell models (cell lines used for proliferation studies)^34^ HeLa nuclei/biochemical preparations; in vitro protein interaction assays^35^	N/A (review)^33^ KD of TAF15 and functional assays (proliferation) plus molecular analyses showing TAF15 regulates a subset of cell-cycle genes via miRNAs^34^ Biochemical and immunoprecipitation assays (pull-down, IP, UV cross-linking) showing direct interaction between TAF15 and U1 snRNP components^35^	33, 34, 35
CEBPB	Modulates Sox9 expression during spermatogenesis; it is involved in the regulation of ER stress response, inflammation, and the differentiation of brown adipocytes and osteoblasts. It regulates Wnt/β-catenin signaling and inhibits myogenesis. Additionally, it participates in cytokine signaling, hematopoiesis, gluconeogenesis, and liver regeneration.	CEBPB overexpression/siRNA; WB; qPCR; differentiation assays^36^ Reporter assays; promoter mutagenesis; ChIP-qPCR^37^ Overexpression; RNAi; luciferase; WB^38^ Literature review^39^ Overexpression; CRISPR KO; WB; metabolic assays^40^ Co-IP; luciferase; RNA-seq^41^ ATAC-seq; RNA-seq; CRISPR perturbations^42^	C2C12 myoblasts^36^ TM4 Sertoli cells^37^ HEK293 and cancer cell lines^38^ N/A (Review)^39^ Mouse white adipocytes^40^ Hepatocyte cell lines^41^ Mouse hepatocytes^42^	CEBPB KD restored myogenic markers^36^ Mutation of CRE/CEBP sites abolishes SOX9 promoter activation^37^ CEBPB knockdown rescues Wnt/β-catenin signaling^38^ N/A (Review)^39^ CEBPB KD abolishes SIRT5-induced UCP1^40^ ZHX3 represses CEBPB-driven transcriptional activation of gluconeogenic genes^41^ CRISPR inactivation of sequential TFs blocks enhancer activation^42^	36, 37, 38, 39, 40, 41, 42
CEBPD	Maintains metabolic homeostasis and regulates immune responses, adaptation to hypoxia, cell migration, and keratinocyte differentiation. It also modulates inflammatory signaling pathways, promotes macrophage activation, and contributes to insulin sensitivity and lipid metabolism.	Review of molecular roles of CEBPD in inflammation^43^ Functional assays in macrophages, RNA-seq, cytokine profiling^44^ ChIP-on-chip, promoter analysis, expression assays^45^ Transcriptomics (RNA-seq), cytokine assays, macrophage activation^46^	N/A Review^43^ Mouse macrophages (BMDM)^44^ Human keratinocytes (primary and HaCaT)^45^ WT *versus* Cebpd^-/-^ macrophages^46^	N/A Review^43^ CEBPD siRNA KD to test functional dependence^44^ CEBPD overexpression and siRNA KD^45^ CEBPD KO mice used to identify CEBPD-dependent programs^46^	43, 44, 45, 46
CEBPG	Controls hematopoietic differentiation by promoting granulocyte development and directing immune cell lineage specification under stress conditions.	Functional assays of hematopoiesis in mice^47^ Overexpression, shRNA KD, xenograft assays ^48^	Cebpg^-/-^ mice^47^ AML cell lines (THP-1, HL-60) ^48^	C/EBPγ is dispensable for steady-state and emergency granulopoiesis^47^ CEBPG KD suppressed AML progression both i*n vitro* and in vivo^48^	47, 48
PRDM15	Regulates B-cell lymphomagenesis and glycolysis. Controls key signaling pathways including PI3K/AKT/mTOR, NOTCH, and Wnt/PCP, contributing to metabolic control and developmental signaling under stress or oncogenic conditions.	Loss-of-function models, transcriptomics^49^ CRISPR KO, metabolic assays, RNA-seq^50^ Review of PRDM proteins^51^	Mouse embryos and ESCs^49^ B-cell lymphoma cells^50^ N/A Review^51^	PRDM15 loss-of-function to test dependence of NOTCH and WNT/PCP signaling^49^ PRDM15 CRISPR KO to test survival/metabolic dependence^50^ N/A Review^51^	49, 50, 51
ATOH8	Regulates endothelial cell proliferation, embryonic development, and neurogenesis; also involved in early stages of cell differentiation and endometrial decidualization.	Review of ATOH8 structure, function, and developmental roles^52^ Decidualization assays, BMP2 stimulation, siRNA KD^53^ Overexpression and KD in neurogenesis assays^54^	N/A Review^52^ Human endometrial stromal cells (HESCs)^53^ Postnatal murine neural tissue/primary neural cells^54^	N/A Review^52^ ATOH8 siRNA KD to test dependence during decidualization^53^ ATOH8 loss-of-function to test dependency in neurogenesis^54^	52, 53, 54
ZNF268	Involved in cell proliferation and migration through NF-κB signaling pathway activation, apoptotic and metabolic processes.	Review of KRAB-ZFP signaling in cancer^55^	N/A Review^55^	N/A Review^55^	^55^
ZBTB38	Controls cell invasion and proliferation via Wnt/β-catenin signaling pathway modulation.	Overexpression, siRNA KD, migration/invasion assays^56^	Bladder cancer cell lines (T24, 5637)^56^	ZBTB38 siRNA KD to test dependency for migration^56^	^56^
KLF9	Mediates glucocorticoid signaling responses circadian rhythm regulation. Involved in estrogen (E2) hormone responsiveness.	Glucocorticoid stimulation, RNA-seq, Klf9 KO^57^ Overexpression, promoter assays, pathway analysis^58^ GR induction, ChIP-seq, RNA-seq, siRNA^59^	Zebrafish larvae + mammalian cells (validation) ^57^ Breast cancer cell lines^58^ KBE16 and pulmonary epithelial cell lines^59^	Klf9 KO to test dependence on GR-mediated transcription^57^ KLF9 modulation to test hormone/circadian-dependent transcription^58^ KLF9 siRNA knockdown to test GR-dependent induction^59^	57, 58, 59
TBPL1	TBP-like basal TF that initiates transcription from TATA-less promoters through interaction with TFIIA and TFIIB. Essential for the transcription of ribosomal protein genes and spermatogenesis. Regulates NF1 gene expression and germ cell differentiation.	Literature review; TF structural analysis^60^ KO mice; immunofluorescence; chromatin analysis^61^	N/A Review^60^ Mouse spermatids^61^	N/A Review^60^ Removing Brdt’s first bromodomain alters chromocenter architecture in spermatids^61^	60, 61
ATF5	Regulates cell proliferation, differentiation, homeostasis maintenance, migration, and apoptosis. Regulates erythroid differentiation in fetal liver and controls Wnt/β-catenin pathway activation.	Review; summarizes ATF5 signaling studies^62^ ATF5 overexpression/KD; western blot; migration assays; luciferase reporter^63^	N/A Review^62^ Human bladder cancer cells (T24, UMUC3)^63^	N/A Review^62^ ATF5 KD decreases activation of the β-catenin pathway, showing functional dependence on ATF5^63^	62, 63
ATF3	Controls glucose metabolism, apoptosis, inflammation, immune responses, and hepatocyte differentiation.	Review on ATF3 in metabolism^64^ Review integrating ATF3 roles in inflammation, apoptosis, ferroptosis^65^ Metabolic assays; ATF3 expression studies; glucose/lipid metabolism analysis^66^	N/A Review^64^ N/A Review^65^ Various metabolic cell systems (not one fixed model)^66^	N/A Review^64^ N/A Review^65^ ATF3 influences pathways related to glucose and lipid metabolism^66^	64, 65, 66
NFE2L1	Mediates cellular stress adaptation, proteasome synthesis, autophagy, ERAD, and antioxidant responses.	Review of NFE2L1/NRF1 in stress^67^ Proteasome inhibition; NRF1 activation assays; qPCR; western blot; autophagy assays^68^	N/A Review^67^ Human cell lines (HeLa, SH-SY5Y)^68^	N/A Review^67^ NRF1-dependent induction of p62 and GABARAPL1^68^	67, 68

One of the most widely studied secondary TFs is the CCAAT/enhancer-binding protein (C/EBP) homologous protein (CHOP), due to its prominent role in promoting apoptosis under conditions of severe and unresolved ER stress.^69^ CHOP is primarily induced by ATF4,^70^ although both ATF6^71^ and XBP1s^72^ also contribute to its transcriptional regulation. CHOP is one of the most well-characterized downstream effectors of the UPR, and its role has been previously reviewed.^69^ Therefore, this article focuses on other, less explored secondary TFs regulated by the UPR-TFs, aiming to expand our understanding of the broader transcriptional landscape governed by the UPR.

#### The ATF4 arm of the UPR: Secondary transcriptional responses

**Fig. 2:**
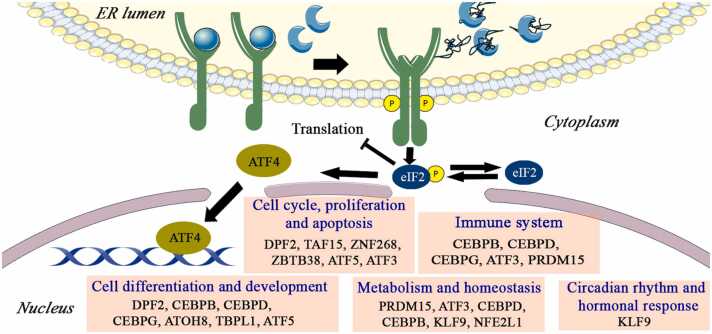
*Activation of the PERK branch of the unfolded protein response (UPR).* Under ER stress, PERK is activated following the dissociation of GRP78, similar to the IRE1α branch. Subsequently, PERK undergoes dimerization and autophosphorylation, enabling it to phosphorylate eIF2α, leading to a general reduction in protein synthesis while permitting selective translation of ATF4. Once in the nucleus, ATF4 induces the expression of secondary transcription factors (TFs) that expand its regulatory network. These TFs can be grouped based on their primary roles: (i) cell cycle, proliferation, and apoptosis (DPF2, TAF15, ZNF268, ZBTB38, ATF5, ATF3); (ii) immune system (CEBPB, CEBPD, CEBPG, ATF3, PRDM15); (iii) cell differentiation and development (DPF2, CEBPB, CEBPD, CEBPG, ATOH8, TBPL1, ATF5); (iv) metabolism and homeostasis (PRDM15, ATF3, CEBPD, CEBPB, KLF9, NFE2L1); and (v) circadian rhythm and hormonal responses (KLF9).

Upon activation of the PERK branch of the UPR, PERK phosphorylates the α-subunit of eukaryotic initiation factor 2α (eIF2α) at Ser51. This modification prevents translation initiation, leading to a global reduction in protein synthesis.^73^, ^74^ However, despite this general translational attenuation, ATF4 mRNA is selectively translated. This selective translation occurs due to the presence of multiple upstream open reading frames (uORFs) within its 5′ untranslated region (Figure 2).

Under normal conditions, ribosomes initiate translation at uORF1 and typically reinitiate at uORF2, preventing translation of the main ATF4 coding sequence. During UPR activation, eIF2α phosphorylation reduces the availability of the eIF2α-GTP ternary complex, thereby delaying reinitiation and allowing ribosomes to bypass uORF2, ultimately enabling ATF4 translation. Additionally, the eIF2D initiation factor, the density-regulated protein, and the malignant T cell-amplified sequence 1 (DENR-MCTS) complex facilitate this non-canonical translation mechanism.^75^, ^76^ Moreover, an internal ribosome entry site located between uORF3 and uORF4 functions as an alternative translation initiation site during the UPR, which further supports ATF4 translation under stress conditions.^77^, ^78^

It is also important to note that PERK is not the only kinase capable of phosphorylating eIF2α. This modification can also occur through activation of the Integrated Stress Response, which involves other kinases, including general control nonderepressible 2, heme-regulated inhibitor kinase, and protein kinase RNA-activated. These kinases are activated in response to various cellular stress conditions, including UV irradiation, amino acid deprivation, ER stress, oxidative stress, mitochondrial dysfunction, heme deficiency, and viral infections.^79^

ATF4 is a basic leucine zipper (bZIP) TF and a member of the ATF/cAMP Response Element Binding (CREB) protein family that forms both homo- and heterodimers through its bZIP domain and contains a DNA-binding domain.^80^ In response to ISR activation, increased ATF4 translation facilitates the recruitment of ATF4-containing transcriptional complexes to C/EBP/ATF response elements (CARE) located within target gene promoters.^81^ Several studies have demonstrated that ATF4 can heterodimerize with TFs, including CCAAT/enhancer-binding protein gamma (CEBPG), Activating transcription factor 5 (ATF5), Activating transcription factor 3 (ATF3), and CHOP.^82^ These diverse protein-protein interactions confer ATF4 with remarkable versatility in regulating distinct gene expression programs depending on the stress stimulus, interacting partner, and cellular context (Figure 2).

Given ATF4′s capacity to form various functional dimers and regulate the expression of downstream TFs,^83^ Table 1 shows a list of secondary TFs regulated by ATF4 and their associated cellular processes.

As illustrated in Table 1, the secondary TFs downstream of ATF4 exhibit diverse functional roles. For example, multiple secondary TFs participate in stress-related responses, with ATF3 regulating glucose metabolism and inflammation, while Krüppel-like factor 9 (KLF9) mediates glucocorticoid signaling and circadian rhythm regulation. Additionally, secondary TFs including Double PHD fingers 2 (DPF2), TATA-box binding protein asssociated factor 15 (TAF15), TATA-box binding protein like 1 (TBPL1), and TFIIB-Related Factor 2 (BRF2) participate in transcriptional regulation and chromatin remodeling, suggesting potential cooperative mechanisms at specific genomic loci to regulate ATF4-dependent gene expression programs.

Taken together, the functional diversity of ATF4-regulated secondary TFs indicates that beyond their roles in alleviating ER stress, these factors regulate diverse cellular processes, including development and differentiation, metabolic homeostasis, immune responses, chromatin remodeling, and stress adaptation. This broadens the repertoire of downstream genes and cellular processes controlled by ATF4, reinforcing its function as a central hub for integrating stress signals with differentiation and metabolic processes (Figure 2).

#### The ATF6 arm of the UPR: Transcriptional adaptation and downstream effectors

**Fig. 3:**
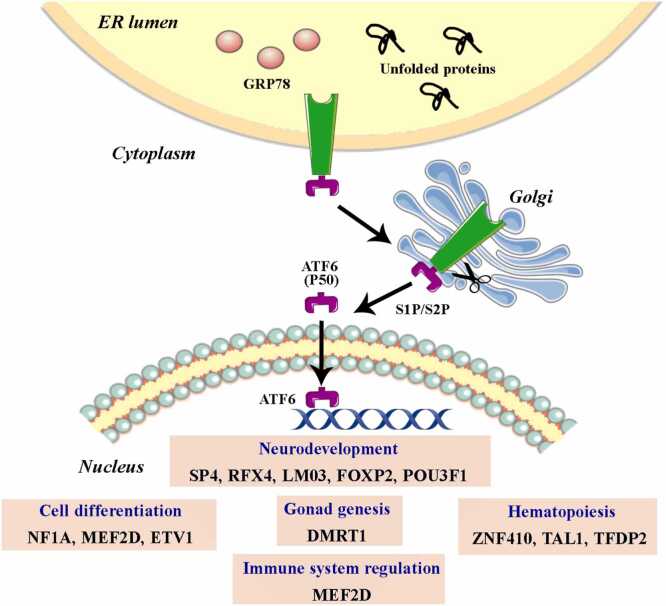
*Activation of the ATF6 pathway in the unfolded protein response (UPR).* When unfolded or misfolded proteins accumulates within the ER lumen, ATF6, a transmembrane protein, is activated and translocated to the Golgi apparatus. There, sequential cleavage by site-1 protease (S1P) and site-2 protease (S2P) releases the active cytosolic domain, ATF6 (P50). This active transcription factor translocates to the nucleus and regulates the expression of secondary transcription factors (TFs). These secondary TFs can be grouped based on their primary roles: (i) neurodevelopment (SP4, RFX4, LM03, FOXP2, POU3F1); (ii) cell differentiation (NF1A, MEF2D, ETV1); (iii) gonadogenesis (DMRT1); (iv) hematopoiesis (ZNF410, TAL1, TFDP2); and (v) immune system (MEF2D).

ATF6 represents another UPR-TF, which is a transmembrane protein with its C-terminal domain residing in the ER lumen and its N-terminal domain extending into the cytosol (Figure 3). The N-terminal region contains a bZIP domain, a DNA-binding domain, and a transactivation domain. Upon UPR activation and subsequent GRP78 dissociation, two Golgi localization signals (GSL1 and GSL2) within the luminal domain become exposed, directing ATF6 to the Golgi apparatus.^84^ In the Golgi, ATF6 is sequentially cleaved by site-1 protease (S1P) and site-2 protease (S2P),^85^ releasing the cytosolic N-terminal fragment that contains the active bZIP domain. This cleaved fragment translocates to the nucleus and functions as an UPR-TF, where it regulates target gene transcription (Figure 3). Notably, ATF6 upregulates several key genes, including GRP78,^86^ the secondary TF CHOP (Yang et al., 2020), and the UPR TF XBP1 (Yoshida et al., 2001), thereby establishing important regulatory feedback loops within the UPR network.

Although mammals express two ATF6 isoforms (ATF6α, with 670 amino acids, and ATF6β, with 703 amino acids), the α isoform exhibits significantly higher transcriptional activity. Both isoforms can form homo- and heterodimers, which enhance their stability during activation. This dimerization facilitates recognition and binding to ER stress-response elements I and II (ERSE I and ERSE II) within target gene promoters.^87^ Given the limited characterization and lower transcriptional activity of ATF6β, this review focuses exclusively on the ATF6α isoform.

To further clarify the downstream regulatory landscape of ATF6α, Table 2 summarizes the secondary TFs that ATF6 influences and their related cellular functions.

**Table 2:** ATF6-regulated secondary TFs and their cellular functions.

Second-ary TFs	Function	Method (used to identify secondary TFs)	Model	Method (used to demonstrate functional role of secondary TFs)	References
TP53	Tumor suppressor that plays a crucial role in maintaining genome stability, regulating cell cycle arrest, apoptosis, and DNA repair.	Review - literature synthesis (no primary method)^88^, ^89^	N/A (review)	N/A (review)	88, 89
HEY1	Involved in embryonic development, the Notch signaling pathway, and epithelial-mesenchymal transition (EMT). Participates in TP53 activation during apoptosis induction and regulates the development of bone, heart, neural tissue, and muscle.	ChIP, reporter assays, RT-qPCR, IP^90^ *In vivo* genetic manipulation, in situ hybridization^91^ Proteomics (MALDI-TOF), mutagenesis, reporter assays^92^ *In vivo* electroporation, transfection assays, reporter assays^93^ Transgenic overexpression and phenotypic analysis^94^ Overexpression and siRNA KD, qPCR and western blot^95^	C2C12 myoblasts, 10T1/2, 293 T cells^90^ Mouse embryos (transgenic/misexpression)^91^ U2OS and cultured cell lines^92^ Mouse embryonic brain and cultured cells^93^ Transgenic mice, bone tissue^94^ Brain microvascular endothelial cells (BMVECs), ischemia animal models^95^	Overexpression/misexpression, ChIP showing reduced MyoD promoter binding^90^ Forced expression (misexpression) and marker analysis^91^ Phosphomutant analysis, co-IP, reporter assays^92^ Electroporation misexpression and inhibition assays^93^ *In vivo* overexpression phenotype; histology and marker analysis^94^ Overexpression and NOTCH3 silencing (siRNA) with rescue experiments^95^	90, 91, 92, 93, 94, 95
ESRRB	Plays a key role in placenta formation, trophoblast development, and interactions with pluripotency TFs and hormones, such as E2 and progesterone (P4).	FUCCI reporters, scRNA-seq, ChIP-seq, enhancer analysis^96^ Mass spec interactome, RNA-seq, ChIP-seq^97^ miRNA arrays, small RNA-seq, integration with ChIP-seq^98^ ChIP-seq, RNA-seq, mutational analysis^99^	Mouse ESCs with FUCCI, ESRRB WT/KO lines^96^ Mouse trophoblast stem (TS) cells^97^ Mouse ESCs with inducible Esrrb^98^ iPSCs and trophoblast-like stem cells^99^	ESRRB KO, overexpression, ChIP-seq and differentiation assays^96^ ESRRB depletion and rescue; interactome profiling^97^ Doxycycline-inducible ESRRB downregulation; ChIP-seq overlap^98^ ESRRB overexpression and domain mutagenesis^99^	96, 97, 98, 99
SP4	It plays a critical role in neural differentiation and dendritic patterning by modulating genes involved in synaptic connectivity, neurite outgrowth, and activity-dependent maturation.	Expression analysis, loss/gain-of-function in mice and cultured neurons; microarray referenced^100^ ChIP, gene expression analysis^101^ Expression profiling, ChIP, reporter assays^102^	Mouse cerebellar granule neurons (in vivo and in vitro)^100^ N2A cells, neurons^101^ Cerebellar granule neurons, mouse models^102^	SP4 mutant mice, rescue experiments, activity-dependent assays^100^ ChIP binding; loss/gain assays^101^ Loss/gain experiments, rescue^102^	100, 101, 102
ZIC4	Participates in neural development. Functions as a potential tumor suppressor, as its downregulation is associated with certain cancers.	Methylation profiling, ChIP for H3K27me3^103^ *In situ* hybridization, expression profiling^104^ Methylation arrays, expression and IHC^105^ Lineage tracing, genetic mutants^106^	HCC cell lines, patient samples^103^ Xenopus embryos^104^ Patient samples, cell lines^105^ Mouse embryos^106^	EZH2 KD/inhibition^103^ Morpholino KD/overexpression^104^ Epigenetic editing/KD and functional assays^105^ Pax6 loss-of-function genetic analysis^106^	103, 104, 105, 106
DRGX	Required for nociceptive sensory neuron development and implicated in neuropathic pain through regulation of pain-related genes during embryogenesis, and contributes to the molecular identity of dorsal root ganglia neurons.	Expression profiling, qPCR, in situ hybridization^107^ scRNA-seq, spatial transcriptomics, bioinformatics^108^	Rat DRG neurons, neuropathic models^107^ Human fetal samples, organoids^107^	In vivo KD/overexpression and behavioral assays^107^ Perturbation in organoids and GRN inference ^107^	107, 108
HMX2	Essential for the development of the inner ear, particularly the vestibular system. It regulates the morphogenesis and patterning of sensory structures derived from the otic placode, including semicircular canals and vestibular ganglia.	Review synthesis of multiple model studies^109^ Morpholino KD, in situ hybridization ^110^ Targeted gene disruption (mouse KO), in situ hybridization, expression analysis^111^	Various (mouse, chick, zebrafish)^109^ Zebrafish embryos^110^ Mouse embryos (otic/vestibular tissues)^111^	Summarizes genetic loss/gain manipulations^109^ Morpholino KD and rescue^110^ Loss-of-function Hmx2 null mice and phenotypic/marker analysis^111^	109, 110, 111
POU3F1	It is a crucial regulator during the transition from pluripotent cells to neural fate. Promotes neural differentiation by activating neural lineage genes and repressing BMP/Wnt signaling pathways.	Review of primary studies (western blot, qPCR, ChIP reported in cited papers)^112^ ChIP-seq, RNA-seq, loss/gain-of-function in ESCs, reporter assays^113^ Genome-wide ChIP-seq and RNA-seq integration; bioinformatic analysis^114^ Review/synthesis of genome-wide chromatin, methylation and histone modification studies (bioinformatic meta-analysis)^115^	Various cancer cell lines (as reported in primary studies)^112^ Mouse embryonic stem cells (ESCs), epiblast stem cells (EpiSCs), neural progenitors in vitro^113^ Mouse ESCs/EpiSCs undergoing neural commitment in vitro^114^ Various embryonic cells (mouse epiblast, early gastrula), ESCs and embryo datasets^115^	Compilation of KD/overexpression experiments from primary literature (shRNA, siRNA, overexpression)^112^ POU3F1 overexpression/KD, ChIP to promoters, neural differentiation assays^113^ Differential expression after Pou3f1 perturbation plus targeted ChIP validation^114^ Summary of primary studies (knockouts, ChIP, ATAC, methylome) rather than new experiments^115^	112, 113, 114, 115
BSX	Regulates synaptic plasticity, long-term potentiation (LTP), and neurocognitive functions, including learning and memory. It acts as a transcriptional regulator in neural circuits involved in cognitive processing. It also modulates neuropeptides involved in energy balance and feeding behavior, and is essential for locomotor activity and metabolic homeostasis. Participates in the differentiation of neuromodulator cell types and integrates neuronal excitability with behavioral and physiological responses.	Chromosomal microarray analysis (CMA) in patients to map deletions and identify candidate genes^116^ Genetic mutants (BSX loss-of-function), expression profiling (RNA-seq, in situ*)*, IHC^117^ BSX KO, behavioral phenotyping, expression analysis^118^	Human patient samples (genomic DNA arrays); no experimental cell models in this study^116^ Zebrafish embryos (BSX mutant and WT); hypothalamic/secondary prosencephalon cells^117^ Mouse BSX null models; hypothalamic tissue analyses^118^	Genotype-phenotype correlation; functional assays not performed in this study^116^ Loss-of-function mutants analyzed for differentiation defects; transcriptional profiling to identify downstream effectors^117^ Loss-of-function (KO) with downstream gene expression and behavioral assays ^118^	116, 117, 118
ZNF410	Important for erythropoiesis and B-cell development in germinal centers.	CRISPR-Cas9 genetic screens in human erythroid cells; ChIP-seq and proteomics mapping^119^ CRISPR-Cas9 screens plus ChIP-seq, proteomics and biochemical genomics ^120^	Human erythroid progenitor/cell line models and primary erythroid cells^119^ Human erythroid cells^120^	ZNF410 KO/KD reduces CHD4 levels and derepresses HbF; rescue and biochemical assays confirm CHD4 mediation^119^ ZNF410 KO/KD and measurement of CHD4 protein and HbF levels; mapping of regulatory elements^120^	119, 120
RFX4	Involved in ciliogenesis during neurodevelopment, regulating the formation and function of primary cilia in neural progenitor cells.	Genetic mouse models, expression analysis, microscopy of cilia, signaling assays^121^ Morpholino KD/CRISPR, in situ hybridization, expression profiling^122^ Comparative genomics, expression analysis in choanoflagellates/related species, functional assays^123^ Review and synthesis of primary studies (ChIP, expression, genetic analyses)^124^	Mouse neural tube/embryonic tissues, cultured cells for ciliogenesis assays^121^ Zebrafish embryos (neural tube) ^122^ Choanoflagellates or related unicellular relatives of animals; comparative species models^123^ Various models depending on cited studies (vertebrate embryos, cell lines)^124^	Rfx4 mutant analysis showing altered Shh signaling correlated with regional ciliogenesis defects^121^ Loss-of-function in zebrafish with midline defects and marker perturbation^122^ Perturbation (RNAi/KO) in unicellular models showing effects on ciliogenesis^123^ Summarizes genetic and molecular experiments from primary literature^124^	121, 122, 123, 124
TAL1	Regulates hematopoietic stem cell differentiation, myeloid lineage specification, and erythroid progenitor development. Participates in the commitment and maturation of blood cell lineages.	Review of genetic and biochemical studies on TAL1 (ChIP, expression, mutational analyses)^125^ Overexpression studies in iPSCs, differentiation assays, flow cytometry, transcriptomics^126^ Isoform-specific expression and functional assays, RNA-seq, KD/overexpression^127^	Hematopoietic cells, cell lines, mouse models (as discussed in review)^125^ Human iPSCs differentiating to hematopoietic lineages in vitro^126^ Hematopoietic progenitors and cell lines; in vitro growth assays^127^	Summarizes evidence from knockouts, overexpression, and mutational studies^125^ TAL1 overexpression and assessment of hematopoietic complex formation and enucleation metrics^126^ Isoform-specific perturbations and assessment of differentiation and proliferation^127^	125, 126, 127
NFIA	Regulates adipocyte differentiation and lineage specification, promoting brown and beige adipogenesis by cooperating with PPARγ, while repressing myogenesis through the regulation of Myod1. Modulates inflammatory and oxidative pathways to maintain metabolic homeostasis and energy balance in adipose tissue.	Genetic manipulation, ChIP-seq, RNA-seq, enhancer analyses^128^ Co-IP, ChIP-seq, transcriptomics, enhancer assays^129^ Review/perspective synthesizing genetic and genomic studies (ChIP, RNA-seq, functional assays)^130^	Adipocyte precursor cells, mouse models, in vitro differentiation systems^128^ Brown adipocytes, precursor cells, mouse models^129^ Various adipocyte models and datasets (review)^130^	NFIA loss/gain experiments with transcriptomic and enhancer activity readouts^128^ NFIA perturbation and co-localization with PPARγ functional assays of brown fat gene expression^129^ Summarizes experimental evidence from primary literature rather than presenting new data^130^	128, 129, 130
ETV4	Involved in endothelial cell survival, migration, and differentiation downstream of Angiopoietin-1/Tie-2 signaling. It contributes to vascular remodeling and angiogenesis by activating pro-survival and pro-migratory gene programs in endothelial cells It is required for hippocampal dendritic arborization and synaptic connectivity, as part of a neurotrophin-responsive network. During development in lung morphogenesis epithelial branching.	*In vivo* genetic deletion and gain/loss of function in neurons; expression analysis; morphological quantification of dendrites^131^ Transcriptomics, KD experiments (siRNA/shRNA), reporter assays^132^ Conditional gene inactivation in lung epithelium, RNA-seq, in situ hybridization^133^ Reporter lines characterization (GFP/RFP), expression mapping, imaging^134^	Mouse hippocampal neurons in vivo and *ex vivo*; cultured neurons ^131^ Human endothelial cell lines in vitro ^132^ Mouse embryonic lung epithelium in vivo; *ex vivo* lung culture^133^ Mouse embryonic tissues; transgenic reporter lines^134^	*In vivo* conditional KO of Etv4/Etv5 and gain-of-function; dendritic morphology and functional assays^131^ siRNA-mediated KD of ETS factors and measurement of Ang-1-induced migration and gene expression; ChIP where available^132^ Conditional Etv deletion leading to branching defects; rescue/epistasis with Fgf10 dosage experiments; expression analyses^133^ Reporter expression in response to FGF10 contexts; imaging and co-localization studies^134^	131, 132, 133, 134
LMO3	It contributes to amygdala and hypothalamic development during embryonic and adult stages, modulating neurogenesis and neuronal differentiation. Functions as a glucocorticoid-dependent regulator of visceral adipogenesis through PPARγ pathway modulation and interacts with p53 to repress its transcriptional activity in adipogenesis and paleostriatum neurodevelopment.	Loss/gain of function in mouse models or in vitro, RNA-seq, in situ^135^ Comparative expression analysis, KD/overexpression, adipogenesis assays^136^ Behavioral phenotyping, transcriptomic profiling (RNA-seq) of amygdala^137^	Mouse developing basal ganglia tissue and neuronal cultures^135^ Human and mouse preadipocytes and adipocyte cultures; in vivo mouse models^136^ Mouse brain tissue (amygdala) from Lmo3-deficient mice^137^	KO/KD and assessment of cell fate markers^135^ LMO3 perturbation and adipogenic differentiation assays showing species-specific effects^136^ Genetic KD and transcriptome analysis linking Lmo3 loss to mood-related gene expression changes^137^	135, 136, 137
MEF2D	Regulates skeletal muscle differentiation, neural survival, synaptic functions, and B-cell-T cell immune synapse formation. Induces myogenic genes expression in cooperation with MyoD and SWI/SNF complexes during muscle development. Promotes neuronal viability and activity-dependent plasticity by enhancing *BDNF* transcription. In the immune system, maintains the germinal-center T follicular helper (Tfh) phenotype in CD4⁺ T cells by modulating cytokine signaling and transcription for adaptive immunity.	Enhancer mapping, ChIP, reporter assays, CRISPR perturbation of enhancer elements^138^ ChIP, RNA-seq, gain/loss-of-function in muscle cells^139^ Genetic models, transcriptomics, ChIP^140^ Conditional perturbation, flow cytometry, RNA-seq, ChIP ^141^ Review synthesis of ChIP, expression, KO studies^142^ RNA-seq, splice isoform analysis, functional assays in muscle cells^143^	Cortical and hippocampal neurons and in vivo models^138^ Mammalian myoblasts and differentiating muscle cells^139^ Early B-cell progenitors in mouse models and cell lines^140^ Mouse immunological models, germinal center B and T cells ^141^ Various neuronal models (review)^142^ Muscle precursor cells and in vivo muscle models^143^	MEF2 perturbation and enhancer assays to show control of Bdnf expression^138^ MEF2 perturbation and transcriptomic analyses showing distinct gene programs^139^ Conditional deletion and expression analysis showing MEF2 requirement for B-cell development^140^ Mef2d KO/perturbation and functional immune assays^141^ Summarizes primary experimental evidence across studies^142^ Isoform-specific perturbation and rescue assays demonstrating necessity for differentiation ^143^	138, 139, 140, 141, 142, 143
ETV1	Essential for circadian rhythm regulation, differentiation of taste receptor cells, and the specification and maturation of cerebellar granule cells and dopaminergic neurons for synaptic integration and excitability.	Activity-dependent induction studies, phosphorylation assays, ChIP^144^ Expression profiling, lineage tracing, KO/KD^145^ Transcriptomics, ChIP, overexpression in cardiomyocytes^146^ Genetic loss/gain in mouse models, transcriptomics, electrophysiology^147^	Cerebellar granule neurons in vitro and in vivo^144^ Mouse taste cell lineages^145^ Rodent and human cardiomyocytes^146^ Mouse heart tissue and cardiomyocytes; human cardiac samples^147^	Manipulation of activity/BDNF signaling and assessment of Etv1 induction and downstream gene expression^144^ Etv1 perturbation and assessment of taste cell differentiation markers^145^ ETV1 overexpression/KD and measurement of conduction-related gene programs and electrophysiology^146^ ETV1 KO and functional cardiac conduction measurements; rescue experiments^147^	144, 145, 146, 147
TSC22D1	Modulates cell proliferation by interacting with c-MYC. Prevents c-MYC binding to CDKN2B and CDKN1A promoters, while promoting TERT activation, functioning as a context-dependent regulator of proliferation and growth suppression.	Molecular biology assays, expression analyses, signaling assays^148^ Reporter assays, ChIP, expression analysis^149^	Cancer cell lines^148^ Cancer cell lines^149^	Overexpression/KD and pathway activity assays showing feedback regulation^148^ TSC-22 modulation and assays of c-MYC activity and downstream gene expression^149^	148, 149
TFDP2	Heterodimeric partner of E2F TF that regulates cell-cycle progression from G1 to S phase, promoting proliferation and inhibiting apoptosis. Modulates cell size during terminal erythropoiesis in cooperation with E2F2. Additionally, inhibits adipocyte differentiation by repressing CEBPA gene expression.	Global transcriptional profiling and inducible factor screening; proteomics^150^ Review and experimental studies summarized (biochemical interaction assays, expression analysis)^151^	Erythroid differentiation models (cell lines and primary cells)^150^ Cancer cell lines and testis tissue (studies compiled)^151^	Perturbation of Tfdp2 and assessment of terminal erythropoiesis markers and function^150^ Co-immunoprecipitation, reporter assays and KD/overexpression from primary studies^151^	150, 151
FOXP2	Regulates neurogenesis, neuronal differentiation, and migration—Modulates neurite outgrowth, synaptic plasticity, and motor skill circuits essential for speech and language development.	Biochemical assays, mass spectrometry, SUMOylation detection^152^ Review of literature (genetic, biochemical, animal models)^153^	Cell lines expressing FOXP2; biochemical systems^152^ Various (songbird models, rodent, human studies)^153^	Mutagenesis of modification sites and assays of FOXP2 activity ^152^ Synthesis of KD/KO and molecular interaction studies from cited works^153^	152, 153
NPAS1	Modulates hippocampal neurogenesis and regulates gene networks associated with neuropsychiatric disorders. Regulates lung branching morphogenesis.	Genetic perturbation and expression analyses^154^ Genome-wide transcriptome profiling, ChIP, in vivo models^155^ Single-cell transcriptomics, lineage tracing, functional assays^156^	Mouse embryonic lung models^154^ Mouse brain tissue and neuronal models^155^ Mouse basal forebrain neurons in vivo ^156^	Loss/gain-of-function and morphological analyses^154^ KO models and transcriptomic analysis showing downstream gene regulation^155^ scRNA-seq identification and functional perturbations (e.g., optogenetics/chemogenetics)^156^	154, 155, 156
ZNF217	Promotes epithelial cell immortalization and proliferation, maintains stem-like characteristics, and enhances tumor growth and metastasis in cancer cells. Suppresses tumor suppressor genes, interacts with HIF pathways, and regulates iron metabolism by downregulating ferroportin, contributing to iron retention and oxidative stress in tumor microenvironments.	Review synthesizing genomic and epigenetic studies^157^ Expression analyses, hypoxia models, stem cell assays^158^ Review of molecular and clinical studies^159^ Hypoxia treatments, RNA methylation assays (m6A), expression profiling (RNA-seq/qPCR)^160^	Various cancer models cited ^157^ Glioblastoma stem-like cells and cell lines ^158^ Various cancer cell lines and patient datasets^159^ Breast cancer cell lines under hypoxia^160^	Summarizes KD/overexpression, ChIP, and epigenetic assays from primary literature^157^ Overexpression and KD with assays for stemness and hypoxia response^158^ Summarizes genetic perturbation, ChIP, and functional assays from literature^159^ KD/overexpression of ZNF217 and ALKBH5; measure pluripotency factor expression and m6A levels^160^	157, 158, 159, 160
E2F8	Regulates cell-cycle progression by acting as an atypical repressor of E2F’s and TPX2 target genes during the G1/S transition and has context-dependent roles in proliferation and inhibition apoptosis in cancer cells	Review/synthesis of ubiquitination and protein-interaction studies (biochemical, proteomic assays)^161^ Structural biology (X-ray), SELEX/HT-SELEX, DNA-binding assays^162^ Review of genetic/molecular studies (ChIP, expression, functional experiments)^163^ Molecular assays, transcriptomics, pathway analysis^164^	Various cell lines (compiled from primary studies)^161^ *In vitro* protein–DNA systems; cell-based validation in cell lines^162^ Various cancer models (reviewed)^163^ Liver cancer cell lines and in vivo models^164^	Summarizes Cyclin F perturbation and ubiquitination experiments from cited literature^161^ Biophysical binding assays and mutational analyses linking structure to DNA specificity^162^ Compilation/synthesis of loss/gain of function, ChIP, pathway analyses from literature^163^ Genetic perturbation (KD/overexpression) of E2F8 and TPX2; assays for glycolysis, angiogenesis, chemosensitivity^164^	161, 162, 163, 164
SREBF2	Regulates cholesterol biosynthesis and uptake with secondary roles in fatty acid metabolism. Essential for lipid homeostasis and involved in development processes like limb patterning and cell survival.	Commentary/review summarizing SREBP pathway evidence^165^ Review of molecular, genetic and physiological studies (ChIP, transcriptomics, metabolic assays)^166^ Molecular cloning, expression analysis, functional assays in mammary cells^167^ Genomic analyses, ChIP, transcriptomics, functional assays in leukemia models^168^	Multiple metabolic models (literature)^165^ Various metabolic cell types and animal models (reviewed)^166^ Buffalo mammary epithelial cells in vitro^167^ Leukemia cell lines and primary patient samples^168^	Summarizes genetic and biochemical studies reported in the literature^165^ Summarizes perturbation studies (genetic/pharmacologic) demonstrating SREBP function^166^ Buffalo mammary epithelial cells in vitro^167^ Perturbation of RORɣ and SREBP2; metabolic and growth assays to show dependence^168^	165, 166, 167, 168
DMRT1	Controls gonad development and maintains somatic and germ cell fate, particularly in male development. Capable of reprogramming ovarian cells toward the Sertoli cell lineage.	Genetic and comparative studies, molecular assays (review)^169^ Molecular assays, KD/overexpression, signaling pathway readouts^170^ Genetic reprogramming; DMRT1 KO/forced expression^171^	Human and animal gonadal tissues / models^169^ Goat male germline stem cells (in vitro)^170^ Ovarian cell populations (mouse, in vivo/*ex vivo*)^171^	KO/KD and cross-species expression analyses summarized^169^ DMRT1 perturbation with downstream TLR4 pathway readouts (qPCR, western blot, signaling assays)^170^ DMRT1 perturbation followed by analysis of sex-specific markers^171^	169, 170, 171
FOXM1	Regulates homeostasis in mitochondria, cell cycle progression (G2/M), DNA replication, and genomic stability; critical during embryogenesis, tissue regeneration, and cellular responses to stress.	Subcellular localization assays; mitochondrial function measurements^172^ Review of cell-cycle gene-expression studies (ChIP, expression profiling, proteomics)^173^ Single-cell transcriptomics under cell-cycle perturbations^174^ Review of FOXM1-regulated signaling pathways in cancer^175^	Human cell lines (cancer-derived)^172^ Various proliferating cell models^173^ Single-cell transcriptomics under cell-cycle perturbations ^174^ Cancer cell models and clinical data^175^	FOXM1 overexpression/silencing followed by assessment of oxidative phosphorylation^172^ Summaries of perturbation experiments affecting cycle-regulated TFs^173^ FOXM1 perturbation followed by single-cell dynamic state tracking^174^ Synthesis of functional studies (FOXM1 overexpression/inhibition)^175^	172, 173, 174, 175

Table 2 summarizes the characterized functions of secondary TFs downstream of ATF6, highlighting their involvement in diverse cellular processes, including metabolism, differentiation, and stress adaptation.^176^

Although not explicitly indicated in the table, it is noteworthy that XBP1s can also regulate some of these secondary TFs, indicating overlapping regulatory networks within the UPR pathways. This overlap is consistent with previous findings demonstrating that specific genes involved in the ERAD pathway require cooperative action of both ATF6 and XBP1s for complete transcriptional activation^177^ (Figure 3).

ATF6-regulated secondary TFs participate in a broad diversity of biological processes, including cell differentiation, organogenesis, neurodevelopment, hematopoiesis and immune system regulation, cell cycle regulation, and gametogenesis, among others (Figure 3). These diverse functional associations suggest that ATF6 influences cell fate decisions beyond ER stress responses, extending to broader developmental and physiological programs. This highlights a potential dual role for ATF6 as both a classical UPR sensor and a master regulator of cell specification and tissue remodeling processes.

#### The XBP1s arm of the UPR: Activation through IRE1-mediated splicing

**Fig. 4:**
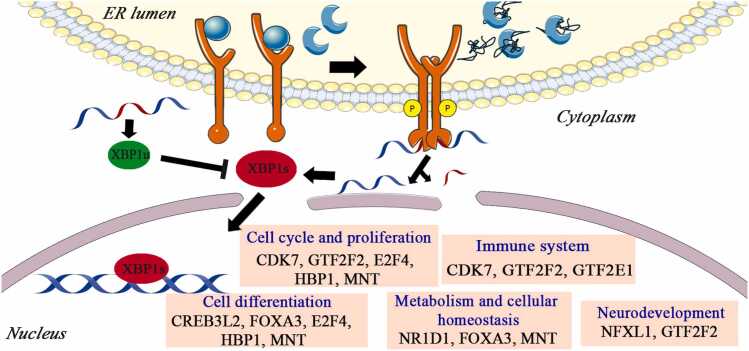
*Activation of the IRE1 branch of the unfolded protein response (UPR).* During ER stress, IRE1α is activated following the dissociation of GRP78 in response to the accumulation of unfolded proteins in the ER lumen. This dissociation triggers IRE1α dimerization and autophosphorylation, activating its cytoplasmic RNase domain. The RNase domain catalyzes unconventional splicing of XBP1 mRNA, producing the active transcription factor XBP1s. Under basal conditions, the unspliced isoform XBP1u predominates and negatively regulates XBP1s activity. XBP1s regulates not only canonical UPR target genes that restore ER homeostasis but also induces secondary transcription factors (TFs) that expand its regulatory scope. These secondary TFs can be functionally categorized based on their primary roles: (i) regulation of cell cycle and proliferation (CDK7, GTF2F2, E2F4, HBP1, MNT); (ii) cell differentiation (CREB3L2, FOXA3, E2F4, HBP1, MNT); (iii) metabolism and cellular homeostasis (NR1D1, FOXA3, MNT); (iv) immune system regulation (CDK7, GTF2F2, GTF2E1); and (v) neurodevelopment (NFXL1, GTF2F2).

Upon ER stress, the transmembrane sensor IRE1α becomes activated through a mechanism similar to that of PERK. The accumulation of unfolded proteins causes GRP78 to dissociate from the luminal domain of IRE1α, enabling its homodimerization and subsequent autophosphorylation. This phosphorylation occurs at key serine residues (Ser724, 726, and 729), which are essential for activating its cytoplasmic RNase domain (Figure 4).^178^

The RNase activity of IRE1α performs an unconventional splicing event on *XBP1* pre-mRNA, removing a 26-nucleotide intron formed by two stem-loop structures (Joshi et al., 2015). This splicing event shifts the open reading frame (ORF), generating a transcript known as *XBP1s*, which differs from the unspliced isoform *XBP1 (XBP1u)* in both exon structure and encoded protein sequence. While *XBP1u* comprises seven exons, the spliced form contains five, resulting in a translated protein that extends from 267 (XBP1u) to 371 (XBP1s) amino acids (Figure 4)^179^.

Both isoforms have distinct C-terminal domains that dictate their cellular functions: XBP1u contains a nuclear export signal and a degradation domain, whereas *XBP1s* harbors nuclear localization and transactivation domains that confer TF activity.^180^ Notably, XBP1u is not produced only in ER stress, acting as a negative regulator of XBP1s during ER stress, preventing its excessive accumulation and transcriptional activity.^181^

Like ATF6, the XBP1s can bind to both ER stress response elements (ERSE I and ERSE II) and the unfolded protein response element (UPRE^26^). XBP1s belongs to the CREB/ATF TF family and contains a bZIP domain, which enables it to form homo- or heterodimers with other TFs, thereby expanding its regulatory capacity. A clear example of this cooperative regulation is the interaction between XBP1s and ATF6, wherein both TFs collaboratively induce the expression of the secondary TF CHOP.^182^ Additionally, XBP1s functions as a positive regulator of ATF4, further supporting the extensive cross-regulation and cooperation among the major arms of the UPR.^183^

To further elucidate the downstream transcriptional network regulated by XBP1s, Table 3 summarizes the secondary TFs transcriptionally induced by this UPR-TF, along with their related cellular functions.^184^

**Table 3:** XBP1-regulated secondary TFs and their cellular functions.

Second-ary TF	Activity	Method (used to identify secondary TFs)	Model	Method (used to demonstrate functional role of secondary TFs)	References
CDK7	Involved in cell cycle progression and acts as a catalytic subunit of TFIIH, a general TF that integrates into the RNA polymerase II pre-initiation complex.	Analog-sensitive CDK7 kinase system; ChIP-seq; mass spectrometry^185^ Transactivation assays; mRNA expression^186^ Structural biology (crystal structure); phosphorylation assays^187^	Human CDK7 analog-sensitive (CDK7as) engineered cells ^185^ Human T-cell lines, PHA-stimulated PB lymphocytes^186^ Purified human CDK7/Cyclin H/Mat1 complex ^187^	CDK7 inhibition (analog-sensitive inhibitor) measuring CTD phosphorylation, Pol II pausing, chromatin marks^185^ HTLV-1 Tax overexpression to activate CDK7 promoter^186^ Mutational T-loop phosphorylation tests to probe activity^187^	185, 186, 187
NR1D1	Regulates circadian rhythms, autophagy, inflammation responses, metabolism, and immune function. It also regulates autophagy and mitochondrial biogenesis, contributing to cellular energy balance and stress adaptation.	Literature review ^188^ KO models; RNA-seq^189^ Review of biochemical regulation^190^	Multiple organ injury models^188^ REV-ERBα/β double-KO mouse ES cells^189^ Liver and other tissues (review)^190^	Summaries of genetic/pharmacological modulation of NR1D1^188^ Genetic deletion of REV-ERVα/β and transcriptomic profiling^189^ Summaries of heme biosynthesis and regulation^190^	188, 189, 190
MNT	Controls cell differentiation, proliferation, and metabolism, functioning as a transcriptional repressor of MYC genes. Acts as a negative regulator of the NF-κB signaling pathway and the EMT.	Functional assays; gene expression; protein interaction analysis^191^ Loss-/gain-of-function; NF-κB pathway assays^192^ EMT assays; expression analysis^193^ Promoter assays; cell proliferation assays^194^	Various cancer cell models (review and experimental) ^191^ Cancer cell lines ^192^ Epithelial cell lines ^193^ MAX-deficient human cell lines^194^	Modulating MNT levels to assess MYC-related pathways^191^ REL (NF-κB) pathway transcriptional assays after MNT perturbation^192^ Overexpression/knockdown of MNT; EMT marker profiling ^193^ MNT KD/overexpression to test autoregulation and growth^194^	191, 192, 193, 194
GTF2F2	Regulates cell cycle, immune responses, and neurogenesis (together with NRF1). Participates in p53 signaling, as well as in TGF-β/SMAD, JAK-STAT, and PI3K-AKT pathways.	Bioinformatics; neurite outgrowth assays ^195^ Bioinformatics: network analysis (WGCNA), gene-expression correlation, enrichment analysis^196^	IMR-32 neuroblastoma cells^195^ Human transcriptomic datasets (depression-related)^196^	NRF1 target gene manipulation to test neurite outgrowth^195^ Correlation of GTF2F2 expression with clinical/depression modules; possible target validation via co-expression^196^	195, 196
CREB3L2	Participates in liver differentiation, regulates secretory pathways alongside Sec23a, and plays roles in chondrogenesis and other developmental processes.	Review/mechanistic summary of ER-Golgi stress, proteolytic cleavage, transcription factor activation^197^ Cell biology, functional assays (ROS, ATP, calcium measurements) Gene expression, transport assays (ER-Golgi), functional activation^198^	Multiple cell types/tissues, as reviewed^197^ Cell lines under ER stress or mitochondrial stress, KO of Creb3 in MEF cells^199^ Hepatic stellate cells (HSCs) in vitro^198^	Multiple cell types/tissues, as reviewed^197^ Genetic or pharmacologic manipulation of CREB3, then measuring Ca²⁺, ATP, ROS^199^ Manipulation of CREB3L2 and measurement of Sec23A/Sec24D mRNA/protein, cell activation markers^198^	197, 199, 198
E2F4	Promotes cell cycle progression and inhibits apoptosis. Represses pluripotency gene expression and contributes to differentiation and quiescence through the DP, RB-like, E2F, and MuvB (dimerization partner, RB-like, E2F, and multi-vulval class B; DREAM) complex. It is also involved in lineage commitment and terminal differentiation.	Genetic KO (CRISPR/Cas9), transcriptomics, ChIP/chromatin interaction^200^ Review/perspective on E2F4′s functions in development and disease^201^ Review of molecular biology of the DREAM complex^202^	Mouse ESCs^200^ Various cell types/model systems (review)^201^ Many cell types (review)^202^	KO of E2F4, then measure effect on gene expression and histone acetylation^200^ Summary of KO, overexpression, genetic studies^201^ Summarizes genetic and biochemical perturbation studies involving DREAM complex TFs^202^	200, 201, 202
NFXL1	Associated with language development and related disorders, including speech and language impairments. Regulates the expression of neural genes involved in communication pathways.	Genetic association/functional genomics^203^ Co-expression gene network/transcriptomic analysis^204^ Review of genetic and molecular studies^205^	Human neural or developmental models (implicated in language impairment)^203^ Embryonic human brain samples^204^ Pediatric populations/developmental disorder contexts^205^	Genetic analysis linking NFXL1 variants to language impairment^203^ Gene co-expression analysis of regulatory genes in brain development and speech-related networks^204^ Summarizes functional and genetic findings in language disorder research^205^	203, 204, 205
ARNT	Controls E2 receptor signaling in response to estradiol and HIF-1α-mediated transcriptional responses to hypoxia.	Molecular medicine/review^206^ Review of AhR/ARNT signaling, gene expression assays^207^ Transcriptomics (global and targeted), molecular biochemistry^208^	Probably various cell and tissue models (HIF-1β/ARNT)^206^ *In vitro* toxicology models^207^ Human T-cell lymphoma cells^208^	Compilation of studies on ARNT regulation, HIF signaling^206^ No specific KD/overexpression assay to test dependence on primary TF^207^ Ligand-dependent manipulation of ARNT isoform ratio; site-directed mutation; phosphorylation assay: they suppressed ARNT isoform 1 or 3, and mutated CK2 phosphorylation site to show dependence on that site for AhR target-gene regulation^208^	206, 207, 208
HBP1	Interacts with chromatin remodelers and activates DNMT1, inhibits cell cycle progression, regulates the ROS pathway, and promotes cellular differentiation (monocytes, adipocytes, keratinocytes).	Review of cell-cycle transcription factors, chromatin binding ^209^	Various normal and cancer cells ^209^	N/A (review) ^209^	^209^
FOXA3	Regulates metabolic homeostasis, lipid synthesis, steroid synthesis in males, and embryonic processes, including pancreas development, adipocyte differentiation, and gluconeogenesis; also functions as a pioneer factor that shapes chromatin accessibility.	Review of genetic and biochemical studies, chromatin assays ^210^ Reporter assays, promoter assays, overexpression ^211^ Adenoviral delivery, siRNA delivery, KO mice were used to crease FOXA3 gain- or loss-of-function models^212^ Review of FOXA family in metabolism^213^	Embryonic development models (in vivo/in vitro)^210^ MA-10 Leydig cells^211^ Liver human biopsies, liver cells (ER stress)^212^ Multiple tissues (review)^213^	*In vivo* and i*n vitro* pioneer factor assays: they show FOXA binding to silent chromatin, opening it, and recruiting other factors to initiate gene regulation^210^ FOXA3 overexpression represses Nur77 promoter activity and downstream steroidogenic gene promoters in a cAMP-induced context^211^ Mechanistically, ChIP-Seq analysis revealed that FOXA3 directly regulates Period1 (*Per1*) transcription, which in turn promotes the expression of lipogenic genes, including *Srebp1c,* thus enhancing lipid synthesis^212^ N/A (review) ^213^	210, 211, 212, 213
GTF2E1	Subunit of the general TF TFIIE, involved in recruiting TFIIH and stimulating its ATPase activity. Alterations are linked to atherosclerosis, colorectal carcinoma, and reduced B-cell populations.	Transcriptome (RNA-seq) analysis ^214^	Human colorectal carcinoma cell line HCT116 ^214^	siGTF2E1 KD of the HCT116 cells and their nontarget negative siRNA controls using RNA-seq^214^	^214^

The secondary TFs regulated by XBP1s, like those controlled by other UPR-TFs, perform diverse functions beyond the UPR response (Figure 4). These include components of the general transcription machinery, such as subunits of the RNA polymerase II-associated factors (CDK7, GTF2F2, or GTF2E1), regulators of metabolism and cellular homeostasis (FOXA3, NR1D1, and MNT), and factors involved in embryonic development (CREB3L2, FOXA2, and NFXL1).

This broad functional diversity demonstrates how XBP1s, through its downstream secondary TF networks, broaden UPR influence to essential cellular processes, including transcriptional regulation, metabolic adaptation, and developmental reprogramming (Figure 4). Additionally, it suggests potential crosstalk and coordination with other UPR-TFs, emphasizing the integrated nature of UPR control over cell fate decisions and cellular homeostasis maintenance.

While this section focuses on secondary TFs regulated by the IRE1α/XBP1s axis, we cannot rule out potential post-transcriptional regulation through the regulated IRE1α-dependent decay pathway, which may modulate mRNAs encoding these secondary TFs or their downstream targets.^22^ The potential role of regulated IRE1α-dependent decay in regulating secondary TF expression warrants future investigation.

Taking this integrative perspective, it becomes evident how each UPR-TF activates distinct secondary TFs with specialized roles. While some of these factors converge on shared cellular processes, others exhibit unique or lineage-specific functions. Collectively, many of these secondary TFs collaborate to alleviate ER stress, as extensively documented in the literature. Overall, this highlights a more comprehensive and interconnected view of UPR signaling, which extends beyond canonical stress responses to encompass complex regulatory networks governing cell fate determination and adaptive responses.

### Alternative modes of UPR activation and their potential impact on secondary transcription factor regulation

Accumulating evidence indicates that UPR activation, and consequently secondary TF activation, can occur through non-canonical mechanisms independent of ER stress. For instance, VEGF activates ATF6 and PERK via PLCγ-mTORC1 signaling, promoting endothelial survival and angiogenesis.^215^ Similarly, in macrophages, toll-like receptor engagement increases reactive oxygen species production through the NADPH oxidase NOX2, leading to selective IRE1α activation and XBP1s induction without engaging other canonical ER-stress markers, thereby enhancing cytokine synthesis efficiency.^216^ Additionally, other studies have demonstrated that UPR sensors can be activated by non-classical triggers to induce cytoprotective autophagy.^217^ These non-canonical UPR activation modes not only bypass the requirement for unfolded protein accumulation but also link UPR signaling to cell survival and lineage-specific functions, which may be mediated by the secondary TFs regulated by the UPR-TFs.

Another emerging non-canonical mechanism of UPR activation is the transmissible ER stress. In this process, cells undergoing UPR activation release extracellular signaling molecules that induce UPR in neighboring recipient cells. Studies in tumor microenvironments have demonstrated that transmissible ER stress signaling can occur through pattern recognition receptors such as TLR4 on myeloid cells, promoting cell survival and drug resistance.^218^, ^219^ Although this phenomenon has been characterized primarily under pathological conditions, it raises important questions about whether this mechanism operates during physiological processes and whether it influences the specific repertoire of secondary TFs activated in recipient cells.

This reciprocal relationship between UPR signaling and the cellular microenvironment has been particularly explored in tumor contexts,^217^ where UPR activation regulates the expression of extracellular matrix (ECM) components and cell adhesion molecules. Within this context, some secondary TFs exhibit functions that extend beyond stress adaptation, directly modulating intercellular communication and tissue architecture. For example, the secondary TF SREBP2 regulates the transcription of genes coding for secreted proteins such as transferrin, thereby modulating iron bioavailability and paracrine signaling pathways.^220^ Similarly, ATF3 controls the expression of matrix-remodeling metalloproteinases (MPP) such as MMP1, MMP2, MMP9, and MMP13.^221^

Although secondary TFs act downstream of the UPR-TFs, accumulating evidence suggests that their transcriptional outputs can indirectly influence upstream UPR activation. By regulating secreted factors, ECM remodeling, and paracrine signaling pathways, some of these secondary TFs can modify the extracellular environment in ways that trigger the UPR activation in neighboring cells. This raises the possibility of a positive feedback loop wherein UPR-regulated secondary TFs indirectly promote subsequent UPR activation. However, comprehensive experimental validation is needed to confirm this model and to systematically identify which secondary TFs possess these regulatory functions.

## Secondary TFs controlled by UPR-TFs: Their roles across organs and development

**Fig. 5:**
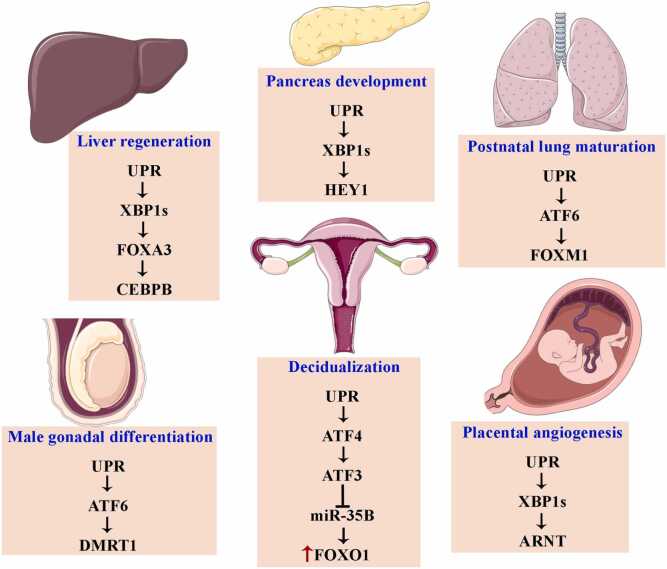
*Organ-specific functions of unfolded protein response (UPR)-regulated secondary transcription factors (TFs).* Beyond their canonical roles in ER stress responses, secondary TFs regulated by UPR-TFs execute diverse developmental and physiological functions across multiple organ systems, including the liver, pancreas, lung, and reproductive organs (gonads, uterus/endometrium, and placenta).

During cellular differentiation and fate determination, transcriptional programs are initiated wherein UPR-TFs function as part of the primary regulators orchestrating context-dependent responses.^222^, ^223^, ^224^ These responses encompass not only canonical UPR target genes but also the induction of distinct secondary TFs. To gain a more comprehensive understanding of these secondary TFs, it is essential to examine their activities across different organ systems and physiological contexts. This approach provides a comprehensive perspective on their contributions to cellular processes that extend beyond canonical UPR-mediated stress responses (Figure 5). Therefore, UPR-induced secondary TFs execute diverse physiological functions across multiple organ systems and developmental stages. The following sections illustrate how distinct subsets of these previously described secondary TFs contribute to specialized regulatory networks in different organs, tissues, developmental stages, and pathological processes. While these TFs participate in different biological processes depending on the context, their presence in these processes does not necessarily mean that the UPR is the main cause of such activities. This method aimed to offer a wider view of the possible roles of secondary TFs beyond traditional UPR-regulated pathways in selected organs.

### Lung

Under oxidative stress conditions, BRF2 inhibits selenoprotein gene transcription in MRC5 lung fibroblasts, exacerbating oxidative stress and triggering apoptosis.^225^

In the context of cancer, such as lung cell lines A549 and NSCLC, increased BRF2 expression correlates with enhanced metastatic potential.^226^ Conversely, TAL1 expression suppresses the metastatic capacity of lung cells by positively regulating SCN4B, a metastatis-supressor gene in lung adenocarcinoma.^227^

Several secondary TFs also play a role in lung development and differentiation (Figure 5). Nuclear factor I A is expressed in murine lung tissue from embryonic day 11.5, contributing to pulmonary organogenesis.^228^ Interestingly, nuclear factor I A is also implicated in cancer biology, where its expression positively correlates with radiosensitivity in non-small cell lung cancer.^229^ E2F4 also participates in lung development by promoting mesenchymal stem cell differentiation into alveolar type II alveolar epithelial cells.^230^ Additionally, FOXM1 plays a critical role in postnatal lung maturation, where its overexpression can lead to defects in airway development.^231^

These findings demonstrate that UPR-regulated secondary TFs not only respond to pathological stress conditions such as oxidative damage, infection, and carcinogenesis, but also orchestrate developmental and differentiation programs in the lung.

### Reproductive organs

#### Testes

DMRT1 regulates male gonadal differentiation processes in both germinal and somatic cells. Notably, DMRT1 functions as a pioneer TF that facilitates chromatin accessibility for the activation of SOX9-regulated genes (Figure 5).^169^ Similarly, RFX4 participates in spermatogenesis through heterodimer formation with RFX2 and RFX3. Defects in RFX4 have been associated with male infertility.^232^, ^233^ TBPL1 also participates in spermatogenesis by organizing heterochromatic chromocenters during haploid spermatid differentiation. Defects in this TF can disrupt chromatin condensation and sperm development.^61^ ZNF410 is expressed in testicular tissue with an uncharacterized function; however, evidence suggests it may regulate CHD4, a component of the NuRD chromatin remodeling complex and essential for germ cell production and gonadal development.^120^, ^234^ Taken together, these observations highlight how UPR-induced secondary TFs extend their regulatory influence into highly specialized processes, including spermatogenesis and chromatin remodeling within male gonadal tissues.

#### Uterus

CCAAT/enhancer binding protein beta (CEBPB) is associated with epithelial cell proliferation and stromal cell differentiation in the uterus, processes regulated by E2 and P4 receptors and essential for embryo implantation.^235^, ^236^ CREB3L2 is another TF regulated by P4 and implicated in Golgi apparatus reorganization and protein secretion, processes essential for decidualization.^237^, ^238^ Notably, ATF3 promotes decidualization by indirectly enhancing FOXO1 expression through suppression of miR-35B, a known FOXO1 inhibitor. Consequently, ATF3 mutations can result in implantation failures.^239^ ARNT (also known as HIF-1β) is also regulated by sex steroid hormones and has been implicated in pathological conditions such as endometriosis (Figure 5).^240^

#### Placenta

Reduced CEBPB expression in the placenta correlates with decreased invasive capacity of extravillous trophoblasts, altered vascular remodeling, and disrupted immune regulation, ultimately leading to implantation failure and preeclampsia development.^241^ Another key TF is ARNT, which forms heterodimers with HIF-2α to promote trophoblast differentiation, angiogenesis, and vascularization under hypoxic conditions (Figure 5).^242^ ARNT KO mice exhibit defective placental vascularization resulting in embryonic lethality,^243^ as well as increased preeclampsia susceptibility.^244^ Under physiological conditions, ATF3 is associated with villous trophoblast invasion, a crucial process for implantation and placental development. It interacts with AP-1, JUN, and JUNB complexes to regulate cell proliferation, survival, and invasiveness.^245^ Additionally, ATF3 functions as a negative regulator of pro-inflammatory cytokines (IL-6, IL-8, and IL-1β), which in turn enhance the expression and activity of MMP-9, COX-2, and prostaglandins in fetal membranes. Notably, decreased ATF3 expression has been observed in fetal membranes during preterm labor with chorioamnionitis.^246^

#### Ovary

CEBPD is expressed in the ovarian theca and interstitial cells and has been associated with steroidogenesis. Its expression transiently increases from early to pre-ovulatory stages, paralleling luteinizing hormone levels.^247^ In a pathological context, CEBPD is upregulated in theca cells from polycystic ovary syndrome patients compared to women without the disease,^248^, ^249^ suggesting a possible link to follicular dysfunction or altered ovarian steroidogenesis.

Overall, these observations show how UPR-regulated secondary TFs extend beyond ER stress management to actively influencing reproductive physiology at multiple levels. From coordinating epithelial proliferation and stromal differentiation in the uterus to controlling trophoblast invasion and vascular remodeling in the placenta and participating in gonads physiology, these TFs are involved in essential processes supporting fertility and pregnancy (Figure 5).

### Hepato-pancreatic

ATF5 plays a pivotal role in maintaining pancreatic islet function by regulating apoptosis under ER stress conditions, as demonstrated in mouse models. Reduced ATF5 expression has been linked to obesity and metabolic dysfunction, whereas its overexpression induces excessive pancreatic β-cell apoptosis.^250^ Interestingly, ATF5 also exerts distinct hepatic functions, interacting with the constitutive androstane receptor to regulate the CYP2B6 enzyme expression, which is crucial for drug metabolism—a process further enhanced under hepatic stress.^251^

During embryonic development, the secondary TFs HEY1 and CEBPD, along with UPR-TF XBP1s, participate in pancreatic organogenesis. Specifically, HEY1 regulates ductal cell differentiation, whereas XBP1s and CEBPD contribute acinar cell differentiation (Figure 5).^252^ Under pathological conditions, HEY1 and CEBPD exhibit opposing roles in hepatocellular carcinoma: HEY1 promotes metastasis, while CEBPD functions as a tumor suppressor gene.^253^, ^254^

FOXA3 and CEBPB are key factors in liver regeneration, with FOXA3 inducing CEBPB expression (Figure 5). These TFs prevent cancer development by upregulating p53, thereby restricting uncontrolled cell proliferation and growth.^255^ On the other hand, FOXA3 is essential during early pancreatic development by maintaining the pool and identity of pancreatic progenitor cells through the regulation of genes that establish exocrine and endocrine cell lineages.^256^

These examples across pulmonary, gonadal, uterine, placental, hepatic, and pancreatic contexts demonstrate how UPR-induced secondary TFs extend their regulatory influence well beyond canonical ER stress responses (Figure 5). By regulating diverse processes such as tissue development, cellular differentiation, regeneration, and pathological outcomes, these TFs reveal a complex regulatory network that connects cellular stress adaptation with broader physiological demands. Recognizing these multifaceted roles highlights not only the flexibility of the UPR but also suggests that its downstream transcriptional effectors are critical nodes linking stress signaling to organ-specific homeostasis and disease susceptibility.

Considering the secondary TFs described above, it is important to emphasize that although many of their functions across tissues, organs, and physiological and pathological processes are not directly driven by UPR activation, a subset of these secondary TFs have been directly involved in the UPR as downstream effectors of the UPR-TFs in related contexts. These secondary TFs, including ATF3,^257^, ^258^ ATF5,^259^, ^260^, CREB3L2,^238^, ^261^ TP53,^262^, ^263^, ^264^ FOXM1,^265^, ^266^, ^267^ CEBPB,^37^, ^236^, ^268^, ^269^, among others, are transcriptionally activated or modulated during ER stress conditions. Their roles within the UPR encompass regulating apoptosis, cell cycle arrest, redox balance, proteostasis, and transcriptional reprogramming to restore ER homeostasis.

## Interaction network and functional characterization of UPR-induced secondary TFs

**Fig. 6:**
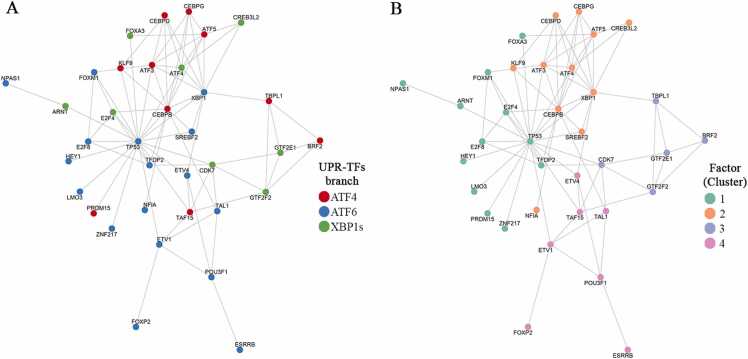
*Interaction network analysis of unfolded protein response (UPR)-regulated secondary TFs.* Network topology showing 35 secondary TFs (nodes) and their functional associations (160 edges) derived from STRING database analysis (A). Node size represents degree centrality, and node color indicates the primary UPR-TF regulator (ATF4, ATF6, or XBP1s). Community detection analysis using the Louvain algorithm identified four distinct modules (color-coded), revealing cross-talk among secondary TFs regulated by different UPR-TF pathways (B).

To elucidate potential interactions among secondary TFs and determine whether these factors exhibit cross-talk across different UPR-TF pathways, we performed interaction network analysis using the STRING platform. The combined list of secondary TFs together with their corresponding UPR-TF regulators was used as input. Network visualization and topological analysis were performed using the igraph and tidygraph packages in R. The resulting TF–TF interaction network (Figure 6 (A)) comprised 35 nodes and 160 edges (representing functional associations). Centrality metrics (degree and betweenness) and clustering coefficients were calculated to characterize the network structure.

To identify functionally related TF clusters, we applied the Louvain community detection algorithm, which groups nodes based on the density of their interconnections. This analysis identified four distinct modules (Figure 6 (B)), each representing a cluster of TFs with strong mutual interactions. The modular organization suggests that TFs within each module likely participate in coordinated regulation of shared biological processes. Notably, secondary TFs within each module were not exclusively associated with a single UPR-TF, indicating that secondary TFs regulated by different UPR-TFs can converge within shared functional networks. These findings support the existence of cross-talk among UPR branches at the level of secondary TFs, potentially enabling coordinated or context-specific transcriptional responses.

### Functional characterization of TFs modules

To characterize the biological functions associated with each module identified through Louvain clustering, we performed Gene Ontology biological process enrichment analysis. This analysis revealed distinct functional profiles for each module, indicating that TFs within each cluster participate in coordinated transcriptional regulation of specific biological processes.

Module 1 exhibited enrichment for developmental and stress-related processes, including embryonic development, morphogenesis, and negative regulation of the mitogen-activated protein kinase pathway . Notably, multiple enriched terms were associated with p53-mediated DNA damage responses, suggesting that TFs in this module participate in cell-cycle control and stress adaptation.

Module 2 showed strong enrichment for canonical UPR-related processes, including ER stress response and UPR activation. Additionally enriched terms included cellular response to nutrient levels and adipocyte differentiation, suggesting that TFs in this module mediate metabolic adaptation and cellular differentiation under stress conditions.

Module 3 was specifically associated with the basal transcriptional machinery and nucleotide biosynthesis. Enriched terms included RNA polymerase II promoter transcription initiation, C-terminal domain phosphorylation of RNA polymerase II, and RNA polymerase III-mediated snRNAs transcription. These findings suggest that TFs in this module regulate core transcriptional processes and maintain transcriptional regulation processes.

Module 4 did not yield significantly enriched Gene Ontology terms, likely due to the limited number of TFs within this module or functional heterogeneity among its members.

It is important to note that not all secondary TFs listed in Table 1, Table 2, Table 3 displayed detectable interactions in the STRING platform or could be assigned to defined network modules. This limitation likely reflects current gaps in protein interaction databases rather than the absence of such interactions. Moreover, many TFs form heterodimeric complexes or participate in multi-protein regulatory assemblies, which may expand their functional connectivity beyond what can be captured by the present analyses. Therefore, experimental validation will be essential to uncover missing interactions and fully characterize the regulatory networks formed by these secondary TFs.

Collectively, these findings indicate that UPR-regulated secondary TFs organize into functional modules associated with distinct biological processes, including development, metabolism, and basal transcription. The presence of TFs from multiple UPR-TF pathways within individual modules supports extensive cross-talk among UPR branches at the secondary TF level. However, experimental validation will be essential to confirm these in silico predictions and comprehensively characterize the functional roles of these transcriptional networks.

## Conclusion

Traditionally, UPR-TFs (ATF4, ATF6, and XBP1s) have been primarily characterized as mediators of ER stress resolution or apoptosis when homeostasis cannot be restored. However, this perspective underestimates the extensive regulatory influence of secondary TFs induced by the UPR-TFs. These secondary TFs orchestrate diverse cellular processes, including differentiation, proliferation, metabolism, neurodevelopment, and cell cycle progression in contexts extending well beyond ER stress responses.

Comparative analysis of the UPR-TFs downstream targets reveals convergence on broad functional and physiological processes. Many of these secondary TFs exhibit tissue-specific activities across diverse organs, including lungs, testes, uterus, placenta, ovary, and liver, where they regulate immune responses, embryonic development, cancer progression, and tissue regeneration. This suggests that UPR-TFs function not only as cellular stress sensors but also as master regulators of transcriptional reprogramming, with roles extending to normal physiological development and tissue-specific homeostasis.

Furthermore, the diverse actions of these secondary TFs across different tissues illustrate how UPR-associated transcriptional pathways can be tailored to specific physiological and cellular contexts. Consequently, UPR-TF activation may determine distinct cell fates depending on tissue environment, developmental stage, or the nature of cellular stress encountered.

Taken together, this evidence reinforces the emerging view that UPR activation extends beyond its canonical role in reducing ER stress and restoring proteostasis, with UPR-TFs functioning as central regulatory nodes within extensive transcriptional networks that exert multi-organ and multifunctional effects. Future investigations will be essential to validate these regulatory connections, elucidate how UPR dysregulation contributes to disease pathogenesis, and comprehensively characterize the roles of other secondary TFs induced by UPR-TFs.

## Funding and support

This research was supported by Instituto Nacional de Perinatología ‘Isidro Espinosa de los Reyes’ (INPer, grant number 2017–2-93), Universidad Nacional Autónoma de México (UNAM-PAPIIT grant numbers IA203822 and IN216024), and PhD scholarship from SECIHTI (CVU 854840). This work was also supported by the Universidad Nacional Autónoma de México Postdoctoral Program (POSDOC).

## CRediT authorship contribution statement

**Miguel Angel Jiménez-Beltrán:** Writing – review & editing, Writing – original draft, Investigation. **Rocío Valle-Bautista:** Writing – review & editing, Writing – original draft, Investigation. **Edgar Ricardo Vázquez-Martínez:** Writing – review & editing, Supervision, Funding acquisition, Conceptualization.

## Declaration of interest

The authors declare that they have no known competing financial interests or personal relationships that could have appeared to influence the work reported in this paper.

## Data Availability

No data was used for the research described in the article.
